# Evaluating the impact of image guidance in the surgical setting: a systematic review

**DOI:** 10.1007/s00464-019-06876-x

**Published:** 2019-06-05

**Authors:** James Dilley, Mafalda Camara, Ismail Omar, Alex Carter, Philip Pratt, Justin Vale, Ara Darzi, Erik K. Mayer

**Affiliations:** Department of Surgery and Cancer, Imperial College London, London, W2 1NY UK

**Keywords:** Image guidance, Clinical, Surgery, Metrics, Framework

## Abstract

**Background:**

Image guidance has been clinically available for over a period of 20 years. Although research increasingly has a translational emphasis, overall the clinical uptake of image guidance systems in surgery remains low. The objective of this review was to establish the metrics used to report on the impact of surgical image guidance systems used in a clinical setting.

**Methods:**

A systematic review of the literature was carried out on all relevant publications between January 2000 and April 2016. Ovid MEDLINE and Embase databases were searched using a title strategy. Reported outcome metrics were grouped into clinically relevant domains and subsequent sub-categories for analysis.

**Results:**

In total, 232 publications were eligible for inclusion. Analysis showed that clinical outcomes and system interaction were consistently reported. However, metrics focusing on surgeon, patient and economic impact were reported less often. No increase in the quality of reporting was observed during the study time period, associated with study design, or when the clinical setting involved a surgical specialty that had been using image guidance for longer.

**Conclusions:**

Publications reporting on the clinical use of image guidance systems are evaluating traditional surgical outcomes and neglecting important human and economic factors, which are pertinent to the uptake, diffusion and sustainability of image-guided surgery. A framework is proposed to assist researchers in providing comprehensive evaluation metrics, which should also be considered in the design phase. Use of these would help demonstrate the impact in the clinical setting leading to increased clinical integration of image guidance systems.

**Electronic supplementary material:**

The online version of this article (10.1007/s00464-019-06876-x) contains supplementary material, which is available to authorized users.

Image-guided surgeries are surgical procedures performed in conjunction with preoperative or intraoperative radiological images derived from a variety of source such as MRI, CT or ultrasound [1]. It continues to evolve as a promising technology innovation, driven by its potential to increase surgical accuracy and safety and augment visualisation of anatomical landmarks and subsurface structures during minimally invasive procedures via laparoscopic, Robotic and NOTES routes. The additional information provided by these systems helps to compensate for a sensory deficit compared with open surgery. Research and development of image guidance has therefore been an attractive topic for the research community, and is demonstrated by the large number of publications over the last 20 years [2].

To date, most research into image guidance systems has focused on the technological performance of the systems themselves. A review paper by Kersten-Oertel et al. [3] evaluating mixed reality image-guided surgery reported that the majority of researchers validated their systems in terms of accuracy as a whole, the registration, the calibration, or overlay accuracy of the real and virtual images. The majority of the systems were not evaluated in the clinical setting or using patients. Only 4% of publications evaluated surgical outcome, just 2% evaluated some aspects of ergonomics and no publication considered the human factor issue of human–automation interaction [3]. The need to consider the three-way relationship between the image guidance system, the surgeon and the patient has been highlighted by Jannin and Korb [4]. Focusing on the engineering rather than the clinical aspect, the authors proposed a theoretical framework based on Health Care Technology Assessment (HCTA) [5] for ensuring rigorous assessment of image-guided interventions that considers the necessary complexity and diversity. Kersten-Oertel et al. [3] also suggested that metrics needed to be developed to capture system resilience; namely, systems are usable under suboptimal, as well as optimal, clinical conditions. The authors highlighted the paucity of psychophysical and human factor studies of visualisation methods and interaction techniques. It was concluded that the future focus of research must now look towards the integration of image guidance systems into the operation theatre and how they aid the surgeon in specific tasks. Although there are surgical specialties and centres using image guidance systems, they are not being used by a majority with any regularity. This, combined with the necessary ethical approval of undertaking studies in the clinical setting, has potentially limited the opportunity to demonstrate the real value of image guidance from a clinical perspective.

A review of the literature was undertaken to determine how the impact of image guidance, in a surgical clinical environment, has been evaluated. The primary objective was to establish what metrics have been used to measure clinical impact of image guidance systems, and what factors, such as high use specialities influenced these. By grouping metrics and employing a simple scoring system, the metrics and factors influencing these could be easily measured and compared. This would allow the comprehensiveness and quality of studies to be rapidly assessed and compared. A secondary objective was to use the metrics identified to build on the framework by Jannin and Korb [4], with a focus on the clinical as opposed to the engineering aspect of image guidance. The proposed framework could then be utilised to design and evaluate future image guidance systems to help promote clinical integration.

## Methods

**Fig. 1:**
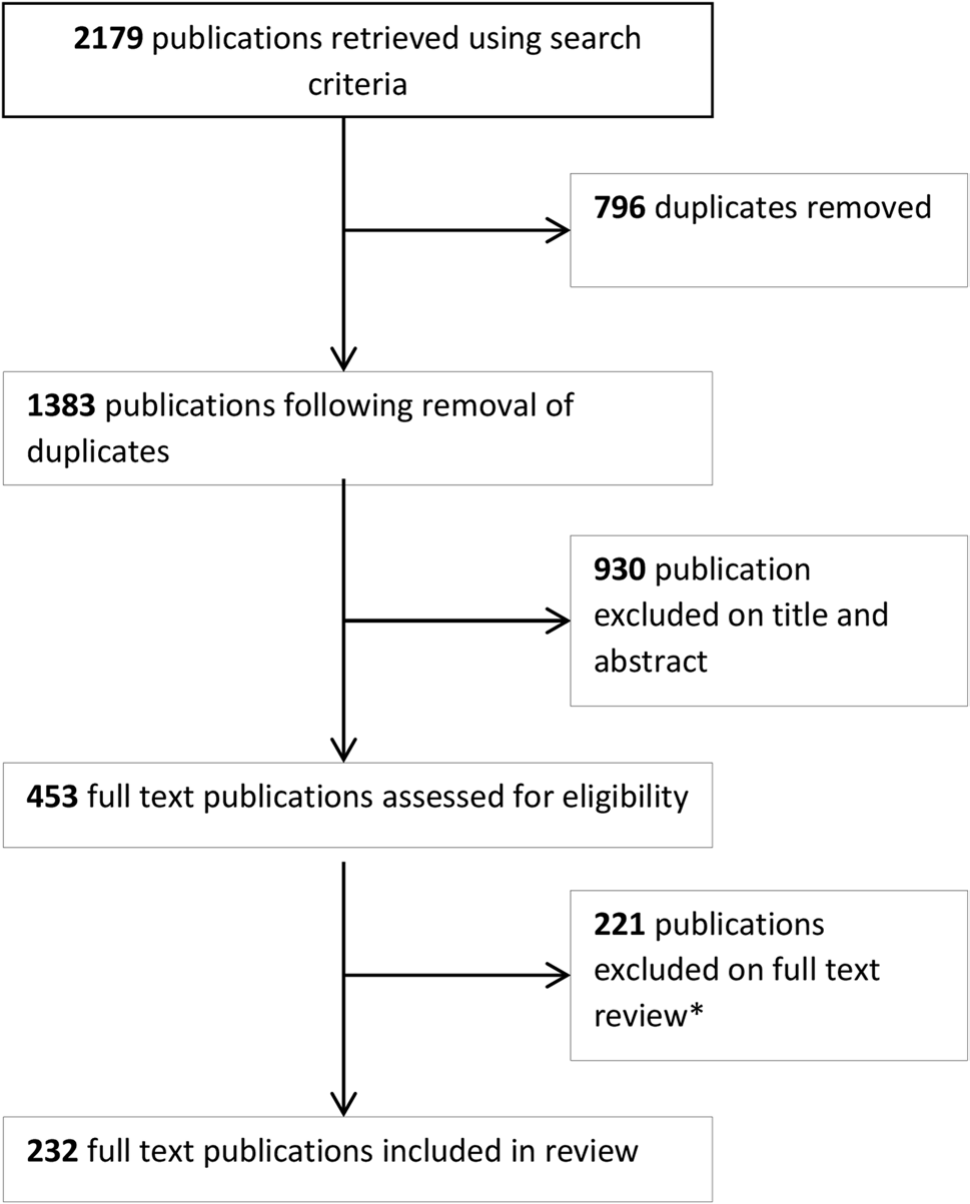
PRISMA algorithm. * Reasons for exclusion (1) The focus was the technical aspect of the system and did not report on clinical translation (this covers just accuracy papers and laboratory studies with non-clinical outcomes); (2) Image guidance was used but not the focus of the paper; (3) used image-free navigation; and (4) case reports of a single case

The preferred reporting items for systematic review and meta-analysis (PRISMA) statement [6] was followed to create the protocol for this systematic review (Fig. 1).

### Search strategy

A systematic search of the literature was performed, using the Ovid MEDLINE and Embase databases, between January 2000 and the end of week 16 2016 (24/4/2016). The search used combinations of the following search terms with appropriate truncations and wildcards through the Boolean operators AND/OR to link them: “augmented reality OR “image guidance”, OR “computer assisted surgery”, OR “mixed visualisation”, OR “mixed reality”, OR “surgical navigation”, OR “image overlay”, AND “surgery”. An English language restriction was applied, and only original articles were reviewed. Two reviewers (JD and MC) independently identified articles that met the inclusion and exclusion criteria, and any disagreements were resolved by a third reviewer (PP).

### Eligibility criteria

Titles and abstracts were screened to identify publications that (1) featured the use of an image guidance system in surgery and (2) described clinical outcomes from its use in the clinical setting. Studies were excluded if (1) they related to radiotherapy or dentistry; (2) needle biopsy and ablative procedures, as these are not always performed by surgeons or in the surgical settings; and (3) if they were conference abstracts, reviews articles and letters.

On review of full papers, studies were excluded if (1) the focus was the technical aspect of the system but did not report on clinical translation (this covers publications solely reporting on accuracy and laboratory studies with non-clinical outcomes); (2) image guidance was used but not the focus of the paper; (3) image-free, molecular or optical navigation was used; and (4) they comprised case reports of a single case.

### Analysis and generation of framework

A data extraction proforma based on previous work by Jannin and Korb [4] and HCTA [5] was created initially using ten papers, read by each reviewer. Relevant themes were identified for inclusion to help ensure all data were captured (Table 1). Publications were screened for the studies’ demographics and methodology, and the remaining data organised into six domains: *system interaction*, *clinical outcome*, *user interaction*, *patient acceptability*, *ethical approval* and *economic impact*. The use of domain alone, to analyse the publications, was considered as too high-level, thereby not providing the granularity needed. For example, a publication could report on each of the six separate domains, but only cover a limited area within each. To overcome the possible limitation with the use of the domain alone, categories were created for each domain. A scoring system, which takes into account the number of categories in each domain, could then be applied to the assessment of each publication: *system interaction* (6), *clinical outcome* (8), *user interaction* (3), *patient acceptability* (1), *ethical approval* (1) and *economic impact* (1), resulting in a total potential category score of 20. Combining the domains and the categories allowed the generation of the proposed framework. (Fig. 4).

**Table 1:**
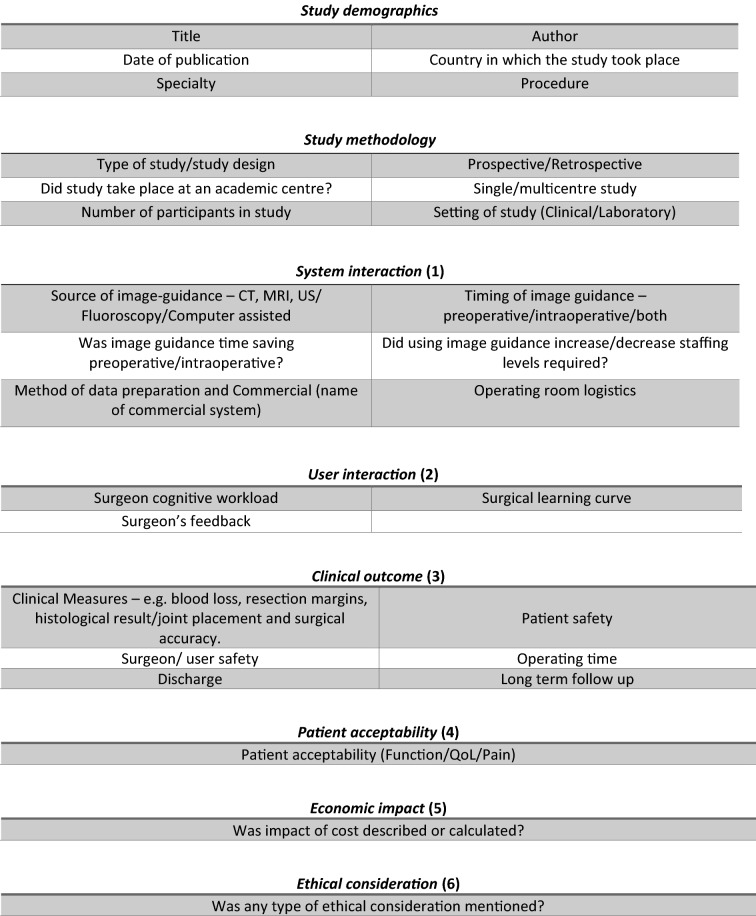
Data proforma summary: categories and respective domains

Population of the data extraction proforma was largely self-explanatory, e.g. ‘procedure’ = ‘craniotomy’, or ‘type of imaging’ = ’MRI’. For those categories requiring a binary Yes/No response, precise details were not always reported, and therefore a categorical classification was used; “Y”, representing being reported and “N” for no reference at all or insufficient evidence. Where analysis involved the year of publication, data from studies published in 2016 were excluded as, due to the timing of the literature search, complete year data were not available.

## Results

### Study demographics and methodology

A total of 232 publications were included in the final analysis (online-only references). Over the period 2000–2016 there was an upward trend in the number of publications. The majority of publications originated from Europe (92), North America (73) and Asia (63). Out of the 232 publications, 195 were performed at an academic centre. Single centre studies accounted for 221 of the publications with only 11 being performed across multiple centres. Fifty-four publications were retrospective and 52 prospective in their study design. Publications covered 14 different specialities; the most common of these were neurosurgery (70 publications), otolaryngology or head and neck surgery (48 publications) and orthopaedics (46 publications).

### Metrics used to evaluate clinical impact of image guidance systems

Of the 232 publications, 227 were carried out in the clinical environment, with five publications exploring clinical impact in a laboratory environment. These five publications focused more on the surgeon in the training and clinical education setting. The types of imaging most commonly used were CT (154) and MRI (62), which were used alone or in combination with an additional imaging modality. In 56 publications, two or more imaging modalities were used. Image guidance systems were used in an intraoperative phase only for 158 publications, the preoperative phase only for four publications and in both phases for 67 publications. The remaining three studies do not present information on the imaging modality used as these were performed in training or clinical education settings.

**Fig. 2:**
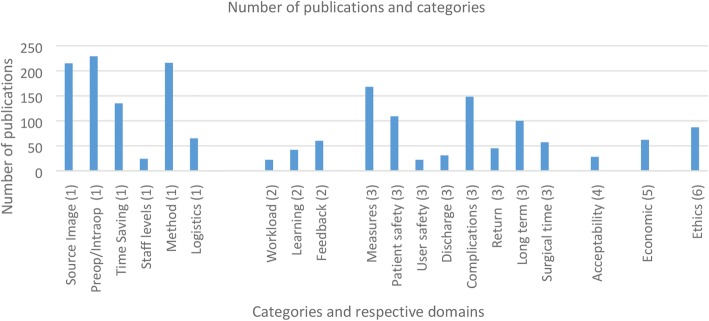
Number of publications as a function of the categories reported on, within each domain. Numbers in brackets indicate which domain each category stems from

The domains most commonly evaluated were *system interaction* (Domain 1) and *clinical outcome (Domain 3)* (Fig. 2). Within the *clinical outcome* domain, the most commonly evaluated categories were clinical measures (168), complications (148) and patient safety (109). The categories of methods of data preparation (216) and timing (229) and source (215) of imaging were the most reported in the system interaction domain. The domains of user interaction, patient acceptability, ethical approval and economic impact were reported less. (see e-Table 1 in the online supplement, which provide further details of evaluated categories within each domain). Details of ethical approval were reported in 87 of 232 publications.

### Analysis of domain and categories score

The most frequent number of domains evaluated was three (104 publications), and the most frequent overall category score was eight (55 publications) with a range of 2–14.

The number of domains evaluated was associated with the total category score obtained; i.e. an increase in the total category score was associated with an increase in the breadth of domains evaluated against, as opposed to one domain being consistently evaluated. Irrespective of the total category score, the domains of *clinical outcomes* and *system interaction* were evaluated most often. *Economic impact* and *user interaction* domains frequency gradually increased in relation to the score.

### Influence of time, volume and methodology on clinical impact

Between 2000 and 2016, the number of publications that evaluated the *clinical outcome* (range 83–100%) and *system interaction* (range 97–100%) domains were consistently high. For the remaining domains, there was no clear trend to suggest either an increase or decrease in reporting over time. Similar findings were observed for the total category score over time. Calculating the mean total category score of the publications in each year showed a range between 6.8 and 9.1, but no clear trend over time. This confirms that there was no increase in either the number of domains evaluated or the total category score over the study period.

**Fig. 3:**
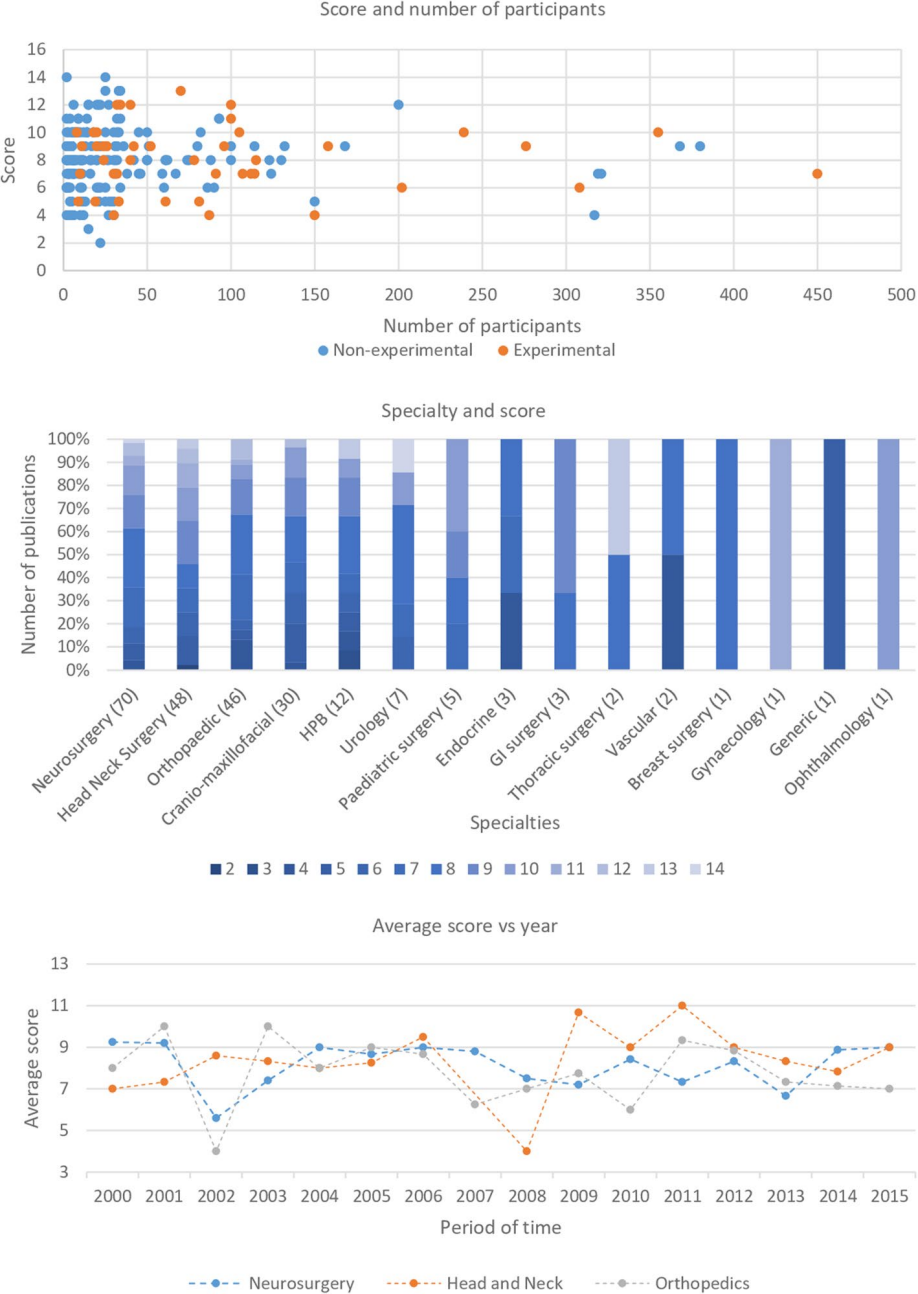
**A** Total category score as a function of the number of participants in each publication, for each type of study design (experimental or non-experimental); **B** Total category score for each specialty, shown as a percentage of publications. Each column represents the score generated for the publications. Numbers in brackets represent total number of publications in each specialty; **C** Mean total category score per year, for the three surgical specialities with the highest number of publications

Publications were categorised as either experimental (randomised controlled trial or case/control) or non-experimental (case series or cohort) in study design. This variable, combined with the number of participants in the publication were compared against their total category score. No correlation (*R*^2^ = 7E − 06) was found between the number of participants and the score of the publication, for either experimental or non-experimental study designs (Fig. 3). There was also no difference in total category score between experimental and non-experimental studies. Both study designs reported similarly in each of the six domains. However, experimental studies did have a slight increase in reporting of ethical approval and patient acceptability.

The uptake and subsequent publications of studies using image guidance are higher in certain surgical specialities (neurosurgery, head and neck surgery, orthopaedics) (Fig. 3). ‘Higher output’ specialties, compared with ‘lower output’ specialities, did neither evaluate more domains nor achieve a higher total category score (see e-Fig. 1 in the online supplement, which shows the Percentage of publications for each specialty grouped by domain). The three highest publishing specialities maintained a consistent total category score over the years, with no increase between 2000 and 2016 (*R*^2^ = 0.024 for neurosurgery; *R*^2^ = 0.0796 for head and neck surgery; *R*^2^ = 0.0223 for orthopaedics) (Fig. 3).

## Discussion

This is the first systematic review paper that establishes what metrics have been used to solely demonstrate the clinical impact of image guidance systems in the surgical setting. The domains most commonly evaluated were *system interaction* and *clinical outcome*. Data preparation and timing/source of image guidance were the categories commonly evaluated within system interaction, and the categories of clinical measures, patient safety and complications within the *clinical outcome* domain. These categories are the most obvious clinical metrics to evaluate, largely because of their familiarity to clinicians and historically have been deemed important when publishing in surgery [7, 8]. Although these categories offer immediate and tangible outcome measures, they also present challenges if being used to demonstrate the true clinical impact of image guidance systems. By focusing on these areas, studies are neglecting to measure important areas, such as the surgeon for whom image guidance was originally intended to assist. Adjustment for this will help in the maturation and evolution of image guidance systems.

The domains of *user interaction*, *patient acceptability* and *economic impact*, although evaluated, [9, 10, 11, 12] were less frequent. These domains have the potential to play a more important role in demonstrating the true impact of using an image guidance system, which in turn will increase their clinical translation [13]. Developing image guidance systems that integrate into existing surgical workflows, whilst achieving the original aim of compensating for a sensory deficit, is more likely to be taken up [2].

### Reporting on high use and developed image guidance systems

One of the objectives of this study was to understand what factors may influences metrics. It was considered that surgical specialities in which image guidance systems were more widely used and better established may have used more comprehensive metrics to evaluate their impact. This appeared, however, not to be the case with similar domain coverage and category scores being achieved for both the highest and lowest publishing specialities, experimental versus non-experimental study designs and no change over the included years 2000–2016.

It may have been assumed that as a technology, such as image guidance, becomes more established, the evaluation of clinical impact would have become more comprehensive over time showing a trend towards a higher category score and/or evaluating across more domains. A possible explanation for the absence of this finding could be that studies are frequently carried out by individual centres, recruiting small numbers of participants and reporting solely on own local work. The lack of information sharing and multicentre collaboration could be considered as a barrier to achieving a more comprehensive evaluation of the technology.

### Generation of a domain and category score

This study has assessed publications using both a domain and a category score aligned with a structure initially proposed by Jannin and Korb [4] to assess image-guided interventions. This study, however, focuses on formalising a set of metrics to enable research to look towards the need to integrate image guidance systems for their sustainable clinical use [3]. The category score was derived to overcome the potential limitation that a publication could report on each of the six separate domains, but only cover a limited area within each. It was found, however, that there was a positive trend between publication total category score and the number of domains covered. This is an encouraging finding, as it suggests that authors who are covering a larger number of domains are aware of the need to cover the breadth of categories within each. The advantage of generating a number or score for a publication is that one can more quickly appreciate the clinical metrics reported and comprehensiveness of the publication. It is important to stress that one should view both the domain and category score, not as interchangeable or independent standards, but rather considered as complementary and thus reported together.

### Limitations

Although this study provides an evaluation of metrics used to assess the impact of image guidance systems in the surgical setting, it is not without limitations. This study employed a broad view of analysing studies using domains and categories in order to successfully established the metrics used to measure the clinical impact of image guidance systems. As such, individual details of studies such as their clinical aims were not observed. Observing this would have been of interest, but the heterogeneous pool of data generated would have distracted from this study purpose. This study stems from the belief that in order for image guidance to become integrated into the surgical workflow, all of the domains involved with image guidance systems need to be observed, not just clinical outcomes. The time period selected for inclusion was only between 2000 and 2016; the year 2000 was adopted because image guidance publications saw a rapid rise in number with regard image guidance development and application from this year [2, 14, 15]. Publications were selected using title search only. The purpose of this review, however, was to search for all publications where the reporting or evaluation of image guidance systems in the clinical setting was the focus. Other reviews of the literature in this field have adopted a similar search strategy, as a result of the volume of publications that would otherwise be obtained [3]. The literature review did not search engineering databases such as IEEE, where image guidance work is published, potentially excluding relevant publications. However, the focus of this review was image guidance systems in the surgical setting. It was anticipated that authors using image guidance systems clinically would publish findings in clinical rather than engineering journals, therefore minimising the potential number of relevant publication missed. The specialities found to be high output are not those traditionally associated with minimally invasive approaches. In these specialties, the anatomy is ‘fixed’, thus reducing a barrier and making it less complicated to generate the image guidance.

### Implications of findings

The translation of novel image guidance technologies into clinical practice requires thorough consideration of the clinical and economic impact of their use. A framework for developing real-world value from a product, if signals for its potential value have been generated from robust studies can facilitate an effective approach to translation. A framework should reflect the component parts of comparative effectiveness and efficiency research on image guidance technologies versus standard(s) of care. A comprehensive research framework should consider all pre-requisites for the translation of these technologies into practice. Outcome-based studies that are categorised as high-quality evidence after formal appraisal and that address all relevant aspects of quality, such as those defined by Donabedian [16], are a pre-requisite. The external validity of studies also requires appraisal because results from this will identify ‘blind spots’ that are relevant to real-world translation. The second component of a value proposition framework therefore refers to the translation of high-quality evidence into normative settings and scenarios. Methods for understanding relevant processes and care pathways that an image guidance technology will ‘disrupt’ should be adopted and added to this framework. This is essential future research. Frameworks in surgical innovation research, such as the IDEAL collaboration exist, which image guidance research should acknowledge and sit within [17]. However, it is felt that the framework proposed in this study builds on the framework proposed by IDEAL providing the granularity required to address the specific issues highlighted in this review to allow diffusion and sustainability in the clinical use of image guidance technology.

**Fig. 4:**
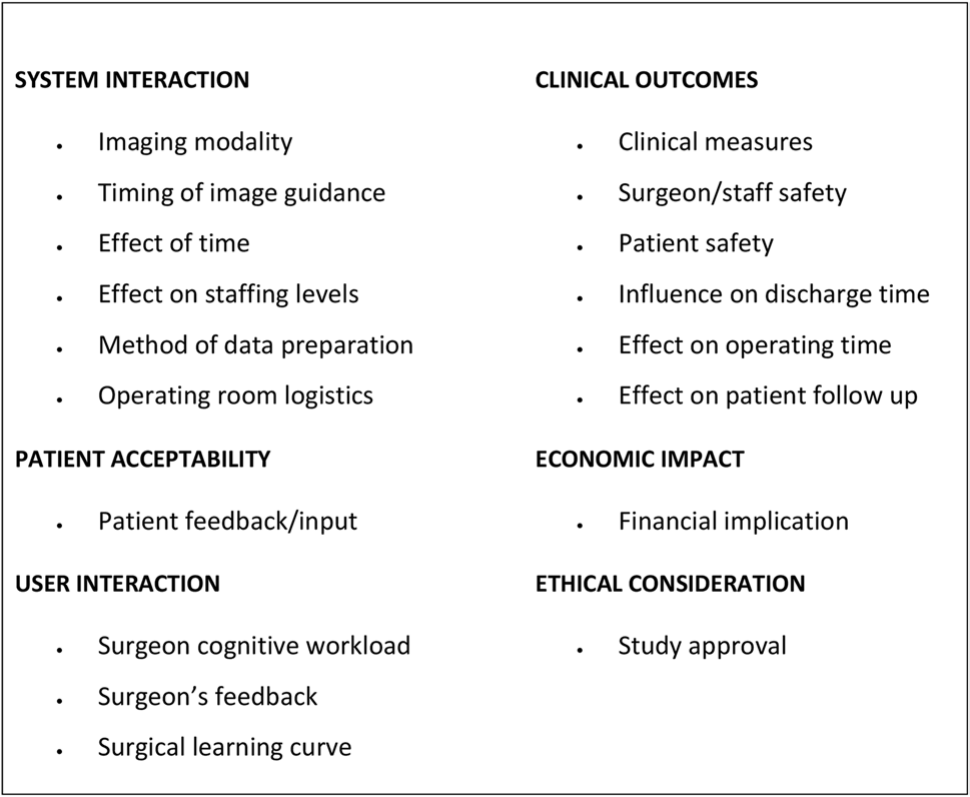
Proposed framework based on domains and corresponding categories

Publications in image guidance have been encouraging research groups to form multidisciplinary teams between the technical and clinical arms to translate these systems into clinical practice efficiently [18]. Although general guidelines have been adopted with this purpose, it has been highlighted that these did not look at the practitioners’ needs [2]. This study has generated a comprehensive view of the metrics that can be reported against and subsequently should be reflected upon by researchers designing studies and reporting on clinical use of image guidance in the future. Based on these, a framework (Fig. 4) is proposed to facilitate researchers in the evaluation of image guidance systems across a wider range of clinical domains, which incorporate the surgeon, staff, patient and infrastructure factors. It is hoped that the framework set out in this publication will be adopted by those publishing work on image guidance. In the meantime, a further review should analyse the change in reporting and adherence to the framework in future publications.

## Conclusion

The findings from this study indicate that publications regarding the use of image guidance systems in the clinical setting are reporting on traditional clinical outcomes but neglecting important areas, such as the surgeon for whom image guidance was originally intended to assist in improving the quality and safety of surgery. It has also uncovered that there has been no evolution in the quality and breadth of clinical metrics despite the many years of use of image guidance in certain specialties. Studies have shown the utility of image guidance systems but in order to evolve, our approach to designing and reporting needs to mature as well. A framework is proposed to act as a catalyst to improving the structure and focus in both design and reporting of image guidance studies to facilitate diffusion and sustainability in this field of surgical research.

## Electronic supplementary material

Below is the link to the electronic supplementary material.

Supplementary material 1 (DOCX 21 kb)

Supplementary material 2 (TIFF 60,449 kb)
