# Supplementary material for: Evaluating the impact of image guidance in the surgical setting: a systematic review
Source: Surg Endosc. 2019 Jun 5;33(9):2785–93. doi: 10.1007/s00464-019-06876-x (PMC6684543; doi:10.1007/s00464-019-06876-x)
Supplement: Supplementary file 1 — Supplementary material 1 (DOCX 21 kb) [file 464_2019_6876_MOESM1_ESM.docx]

| **Domain** | **Category** | **Yes** | **No** | **Total** |  |
| --- | --- | --- | --- | --- | --- |
| **System interaction (Domain 1)** | Impact on workflow time | 126 | 106 | 232 |  |
|  | Impact on staffing levels | 23 | 209 | 232 |  |
|  | Data preparation | 216 | 16 | 232 |  |
|  | Operating room logistics | 58 | 174 | 232 |  |
|  | When image guidance used | **Preoperatively (alone)** | **Intraoperative (alone)** | **Both** | **Not available** |
|  |  | 71 (4) | 225 (158) | 67 | 3 |
|  |  | **Yes** | **No** | **Total** |  |
| **User interaction(Domain 2)** | Surgeon Cognitive workload | 15 | 217 | **232** |  |
|  | Surgical learning curve | 31 | 201 | **232** |  |
|  | Surgeon’s feedback | 57 | 175 | **232** |  |
| **Clinical outcome (Domain3)** | Clinical measures | 156 | 76 | **232** |  |
|  | Patient safety | 59 | 173 | **232** |  |
|  | User safety | 21 | 211 | **232** |  |
|  | Discharge | 24 | 208 | **232** |  |
|  | Complications | 147 | 85 | **232** |  |
|  | Return to theatre | 44 | 188 | **232** |  |
|  | Long term follow-up | 88 | 144 | **232** |  |
|  | Operating time | 56 | 176 | **232** |  |
| **Patient acceptability (Domain 4)** |  | 28 | 204 | **232** |  |
| **Economic Impact (Domain 5)** |  | 29 | 203 | **232** |  |
| **Ethical consideration (Domain 6)** |  | 87 | 145 | **232** |  |

**eTable 1** – Number of publications evaluating each category, within all six domains (System Interaction (Domain 1), User Interaction (Domain 2), Clinical Outcome (Domain 3), Patient Acceptability (Domain 4), Economic Impact (Domain 5) and Ethical Consideration (Domain 6).
