# Supplementary figures and images for: Evaluating the impact of image guidance in the surgical setting: a systematic review
Source: Surg Endosc. 2019 Jun 5;33(9):2785–93. doi: 10.1007/s00464-019-06876-x (PMC6684543; doi:10.1007/s00464-019-06876-x)

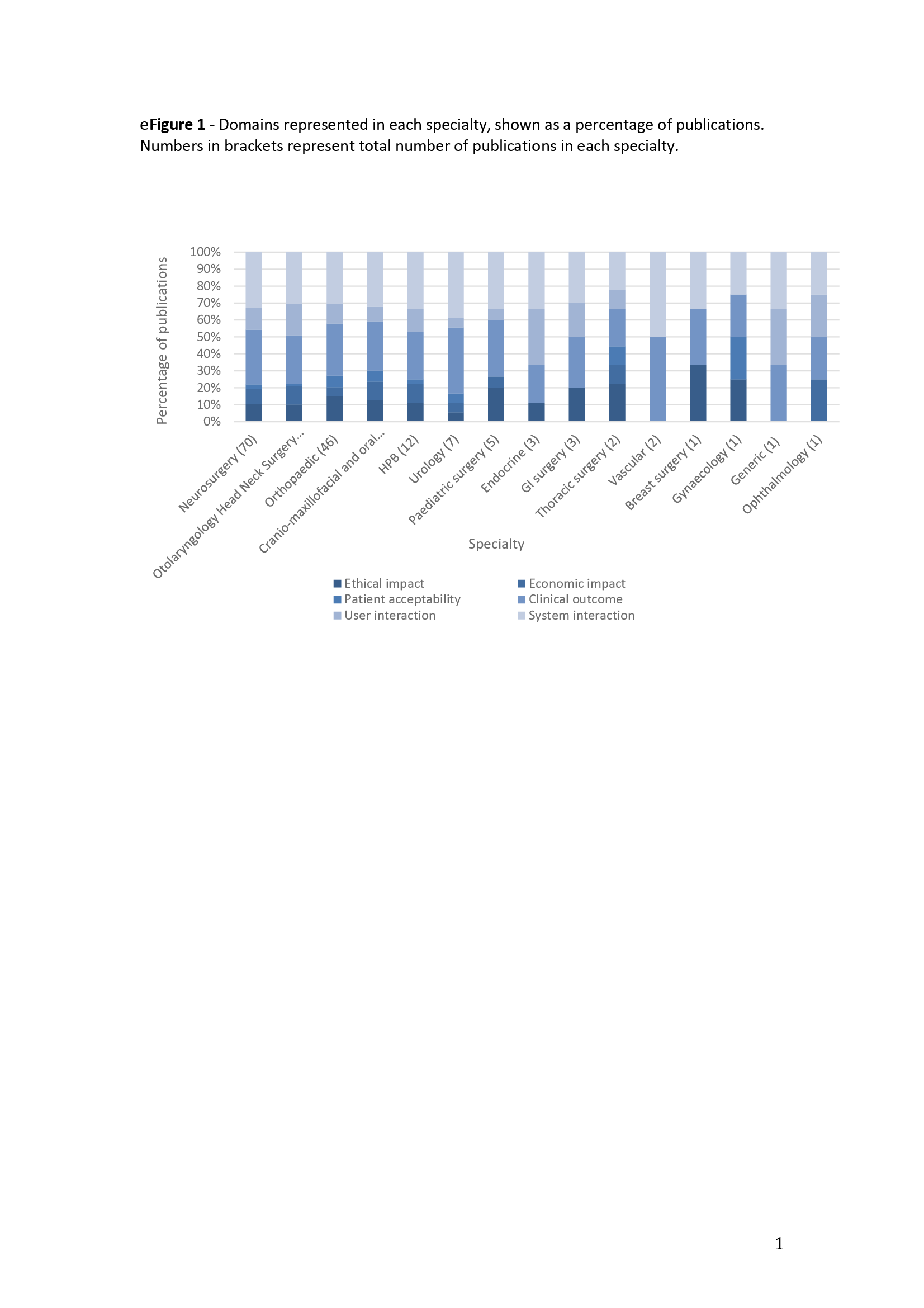

Supplement: Supplementary file 2 — Supplementary material 2 (TIFF 60,449 kb) [file 464_2019_6876_MOESM2_ESM.tiff]
